# Engineering of enzymes using non-natural amino acids

**DOI:** 10.1042/BSR20220168

**Published:** 2022-08-05

**Authors:** Yiwen Li, Paul A. Dalby

**Affiliations:** Department of Biochemical Engineering, University College London, London, Gower Street WC1E 6BT, U.K.

**Keywords:** Directed Evolution, Enzyme engineering, non-canonical, non-natural, Synthetic biology

## Abstract

In enzyme engineering, the main targets for enhancing properties are enzyme activity, stereoselective specificity, stability, substrate range, and the development of unique functions. With the advent of genetic code extension technology, non-natural amino acids (nnAAs) are able to be incorporated into proteins in a site-specific or residue-specific manner, which breaks the limit of 20 natural amino acids for protein engineering. Benefitting from this approach, numerous enzymes have been engineered with nnAAs for improved properties or extended functionality. In the present review, we focus on applications and strategies for using nnAAs in enzyme engineering. Notably, approaches to computational modelling of enzymes with nnAAs are also addressed. Finally, we discuss the bottlenecks that currently need to be addressed in order to realise the broader prospects of this genetic code extension technique.

## Introduction

As biocatalysts, enzymes offer many advantages over traditional chemical catalysts. They are produced by living cells, offer higher catalytic efficiency, and also provide the capability of catalysing reactions in a highly stereoselective manner. Most importantly, as a biodegradable substance, enzymes can replace many polluting catalysts and reduce tons of environmentally unfriendly substances, in line with the concept of ‘green chemistry’ [1]. In recent decades, with continuous technological breakthroughs in enzyme engineering, their application in industry, agriculture, medicine and health, energy development, and environmental engineering have become increasingly widespread. According to a market analysis report, the global biocatalysts market will witness a CAGR of 6.90% during the forecast period of 2022–2029 [2]. All indications showed that there is a very promising market for biocatalysts, and with the development of related technologies, the engineering of enzyme functions, and properties including activity, stereoselective specificity, substrate range, thermal, and solvent stability, etc. has become a hot topic [3].

The main traditional strategies for enzyme engineering include direct evolution, rational design, and semirational design [4,5]. However, the limited ranges of side chains from the 20 natural amino acids limit the possibilities to develop more exotic functionalities. In living organisms, although cofactors or chemical modifications have expanded the range of amino acids to some extent, they still have certain drawbacks. With the development of genetic code expansion (GCE) technology, non-natural amino acids (nnAAs), also known as noncanonical amino acids (ncAAs) or nonstandard amino acids, can be efficiently and accurately translated into proteins *in vivo* and *in vitro* with the help of orthogonal incorporation systems [6–8]. When designing enzymes with artificial residues, the two key points are the choice of substitution sites and the type of nnAAs. Thus, nnAAs and enzyme backbones should first be considered separately. For example, the substitution sites within the scaffold can be selected by traditional protein design methods, considering location, potential impact on stability and function, and any known mutability of the site. The main considerations in selecting potential nnAAs are their chemistry (and potential impact on stability and function), current availability or ease of synthesis, and then the cost and efficiency of their corresponding incorporation systems. The combination of these options might then be considered by the following rules [9]: (1) The replacement nnAA should be structurally similar to the original one. (2) The nnAA is more likely to be successfully inserted into sites close to the N-terminal than the C-terminal of the protein. (3) The introduction of more than two nnAAs into a protein can significantly reduce the yield. In addition, due to the need to use live cells for expression, the inserted nnAAs need to be harmless to the cells and stable. Following these basic principles, numerous nnAAs have been successfully utilised in enzymes to improve their catalytic properties or to develop unique functions over the last two decades [9,10]. In the present review, we have focused on the applications and strategies for using nnAAs in enzyme engineering. Notably, approaches to computational modelling of enzymes with nnAAs are also addressed. Finally, we discuss the bottlenecks that currently need to be addressed in order to realise the broader prospects of using nnAAs in enzyme engineering.

## Orthologous aminoacyl-tRNA synthetase–tRNA pairs: key for the site-specific incorporation of nnAAs

**Figure 1:**
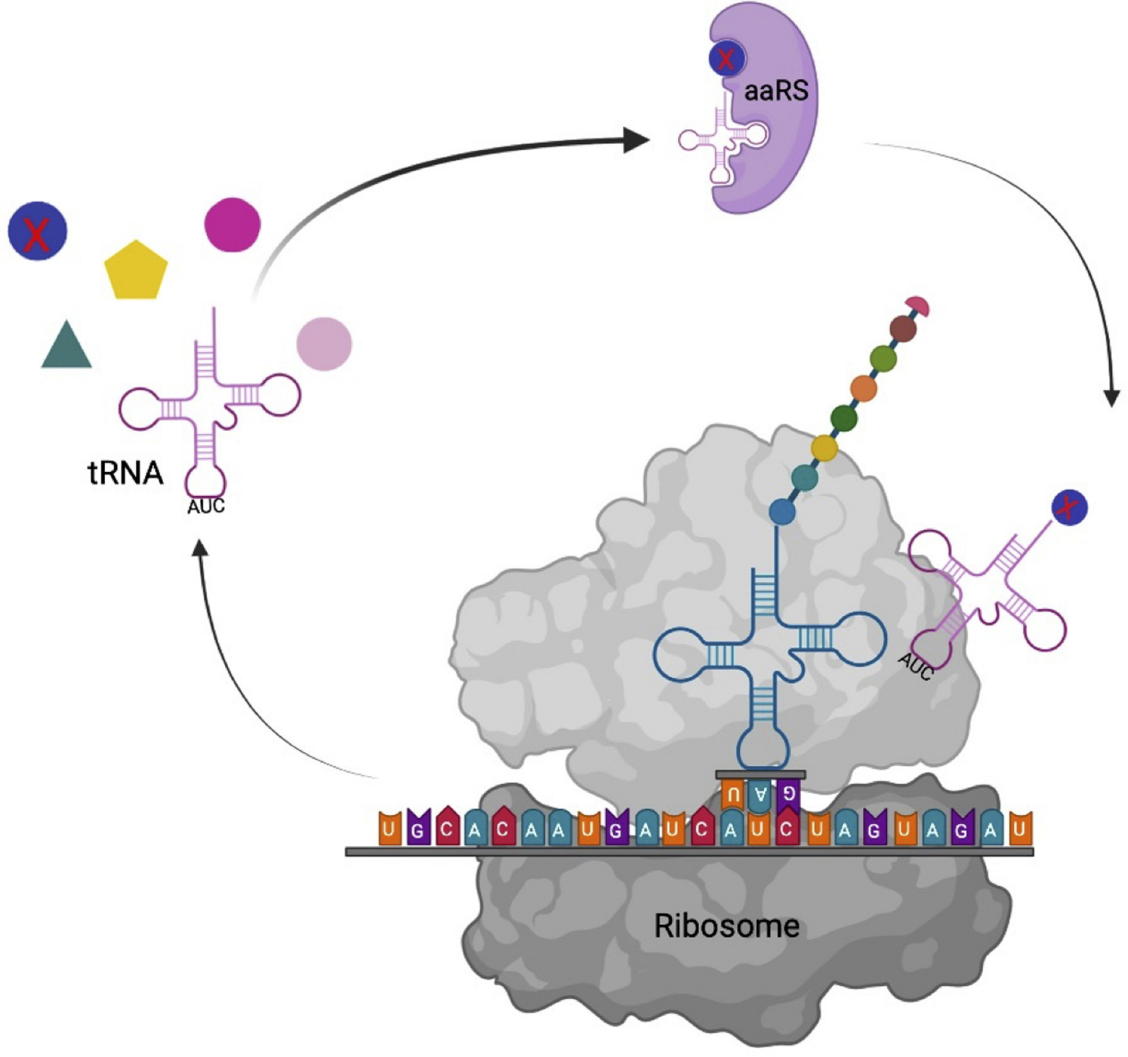
Schematic showing the process of incorporation of nnAAs into proteins via orthologous aaRS–tRNA pairs

To transfer the genetic information on mRNA into the encoded protein through the translation system, engineered tRNAs must only link the desired nnAA but not canonical amino acids to the reassigned codon [11]. New exogenous aminoacyl-tRNA synthetase (aaRS) is also required in this step to aminoacylate only the corresponding tRNA and no endogenous tRNAs. These exogenous elements, including orthogonal aaRS/tRNA pairs, reassigned codons, and nnAAs, form an orthogonal translation system, which means a system with little or no cross-over with the natural biological system (Figure 1). To date, more than two-thirds of nnAAs expressed in *Escherichia coli* (*E. coli*) and mammalian cells have been achieved by adopting *Methanocaldococcus jannaschii (Mj)* TyrRS-tRNA^Tyr^ and *Methanosarcina mazei/Methanosarcina barkeri* PylRS-tRNA^Pyl^ pairs or their evolved variants [11–13]. The former has been shown to introduce more than 100 unnatural side chains in different organisms, mainly phenylalanine and its derivatives, while the latter showed the ability to incorporate more than 50 tyrosine analogues [14,15]. In addition, a growing number of newly developed aaRS/tRNA pairs have been shown to introduce a wider variety of nnAAs, such as LeuRS/tRNA_CUA_ [16], TrpRS/tRNA_CUA_ [17], SepRS/tRNA_CUA_ [18], and LysRS/tRNA_CUA_ [19] pairs. However, they often suffer from small host range, a small variety of suitable nnAAs, and low efficiency; therefore, it is extremely important to evolve more efficient orthologous systems.

Directed evolution provides a powerful tool for screening orthologous aaRS–tRNA pairs with high specificity [20,21]. Target residues within the aaRS active site are mutated to generate libraries, which are then subjected to a two-step screening process over multiple cycles to select mutant aaRS/tRNAs that accept only the desired nnAAs. Based on this method, an increasing number of orthologous pairs have been developed, enabling more than 200 nnAAs to be successfully incorporated into proteins [22]. Using Pyrrolysyl-tRNA synthetase as an example, its high tolerance to substrate side chains and low selectivity towards α-amine and tRNA anticodons allow it to be relatively easily evolved to accommodate more nnAAs or α-hydroxy acids into proteins [23]. Comparing to native PylRS, these engineered variants exhibit a higher efficiency of nnAA incorporation, or can carry lysine and phenylalanine derivatives that the native one does not recognise. Among these variants, the N346A/C348A double-mutant constructed by Liu’s group was shown to act on a wide range of substrates including a series of *p*-alkoxy-Phe, *m*-substituted Phe, and *o*-substituted Phe derivatives in *E. coli* and mammalian cells [24–27]. Notably, the vast majority of evolved aaRS–tRNA pairs only target the amber codon [13], which means that incorporating multiple nnAAs would require the use of alternative codons or new strategies. In general, to further increase the variety of side chains that can be genetically encoded, it is essential to evolve more incorporation systems with better properties.

## Applications

### Improve the activity of enzymes

The use of ‘hotspots’ to guide the engineering of enzymes has gradually grown as a viable and efficient strategy [28]. Changes to structure-based hotspots, including active-site residues, tunnel sites, flexible sites, distal sites coupled to the active centre, and subunit interfacial sites, are highly likely to alter the activity, stability, selective specificity, and other properties of the enzyme. With the widespread use of unnatural amino acid-based techniques, the replacement of these sites with unnatural amino acids has led to a significant increase in the activity of numerous enzymes.

**Figure 2:**
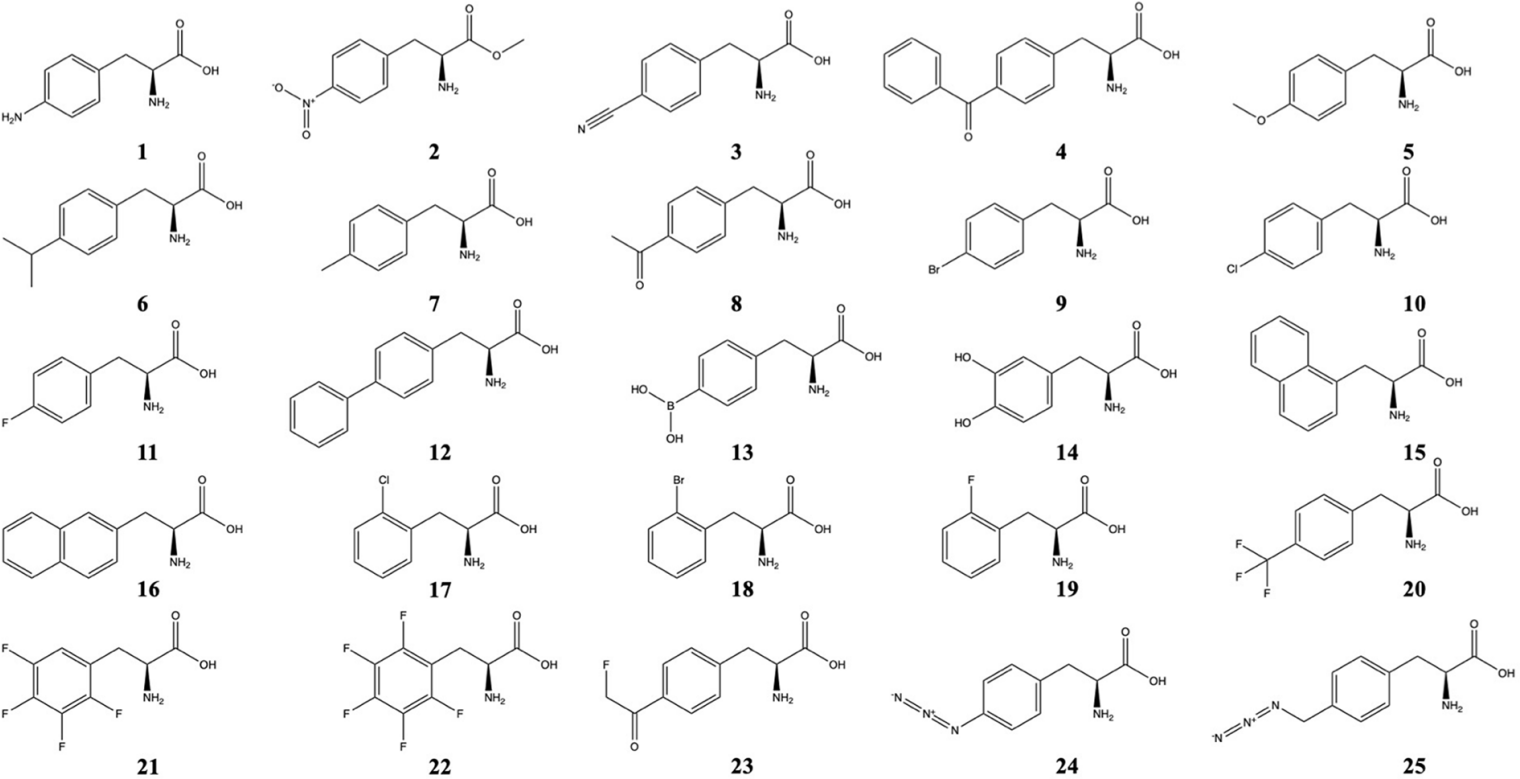
Structures of 25 commonly used phenylalanine derivatives 1, *p*-aminophenylalanine (*p*AMF); 2, *p*-nitrophenylalanine (*p*NTF); 3, *p*-cyanophenylalanine (*p*CNF); 4, *p*-benzoylphenylalanine (*p*BzF); 5, *p*-methyoxyphenylalanine (*p*MOF); 6, *p*-isopropyl-phenylalanine; 7, *p*-methylphenylalanine (pMF); 8, *p*-acetylphenylalanine; 9, *p*-bromophenylalanine (*p*BrF); 10, *p*-chlorophenylalanine (*p*ClF); 11, *p*-fluorophenylalanine (*p*FF); 12, *p*-biphenylalanine; 13, *p*-boronophenylalanine; 14, L-3,4-dihydroxyphenylalanine (DOPA); 15, 3-(1-naphthyl)-L-alanine (NapAla); 16, 3-(2-Naphthyl)-L-alanine; 17, *o*-chlorophenylalanine (*o*ClF); 18, *o*-bromophenylalanine (*o*BrF); 19, *o*-fluorophenylalanine; 20, *p*-trifluoromethylphenylalanine (*p*tfmF); 21, 2,3,4,5-tetrafluorophenylalaine (F_4_F); 22, pentafluoro-phenylalanine (F_5_F); 23, *p*-fluoroacetylphenylalanine; 24, *p*-azidophenylalanine (*p*AzF); 25, *p*-azidomethyl-phenylalanine (*p*AzMeF).

Due to the high incorporation efficiency and ease of synthesis, derivatives of phenylalanine are widely used in enzyme engineering, with 25 commonly used phenylalanine derivatives listed in Figure 2. Transaminases (TAms) are one of the most promising biocatalysts in organic synthesis for the preparation of chiral amino compounds. Replacement of the Phe88 residue in the active site of (*R*)-amine TAm with *p*-benzoylphenylalanine (*p*BzF, **4**) showed a pronounced improvement in the activity towards 1-phenylpropan-1-amine and benzaldehyde [29]. The introduction of a single *p*BzF into the active residue reshaped the size of the active pocket while maintaining hydrophobicity, thus further enhancing the range of substrates.

Another example targeted the aromatic residue Phe385 in transketolase (TK), which has shown its critical role in new acceptor substrate binding. In 2020, this site was replaced with a series of derivatives of phenylalanine (**1**–**3**) on a previously created TK scaffold to reduce the aromatic ring electron density [30]. The results showed that the *p*-cyanophenylalanine (*p*CNF, **3**) mutant simultaneously increased the activity and stability of TK towards 3-hydroxybenzaldehyde (3-HBA), most likely due to the conversion of this functional group from an electron donor to an acceptor, which formed a new hydrogen bond with G262 to stabilise a local helix-turn structure.

*E. coli* nitroreductase (NTR) is being used as a prodrug activator in cancer therapy [31]. Guided by structural information, Phe124 in the active site acts as the key to substrate binding. This site was replaced by eight nnAAs with the help of an evolved *Mj*TyrRS/tRNA^Tyr^_CUA_ pair. Variants incorporating *p*AMF **1**, *p*Bpa **4**, *p*MF **7**, *p*tfmF **20**, and *p*NF-NTR **2** showed better catalytic efficiency towards its substrates CB1954 or LH7. Of these, *p*AF-NTR showed a more than 30-fold increase in activity, probably due to the enhanced π-stacking with polarised aromatic substrates at the 124 site, which also facilitated hydride transfer [32].

**Figure 3:**
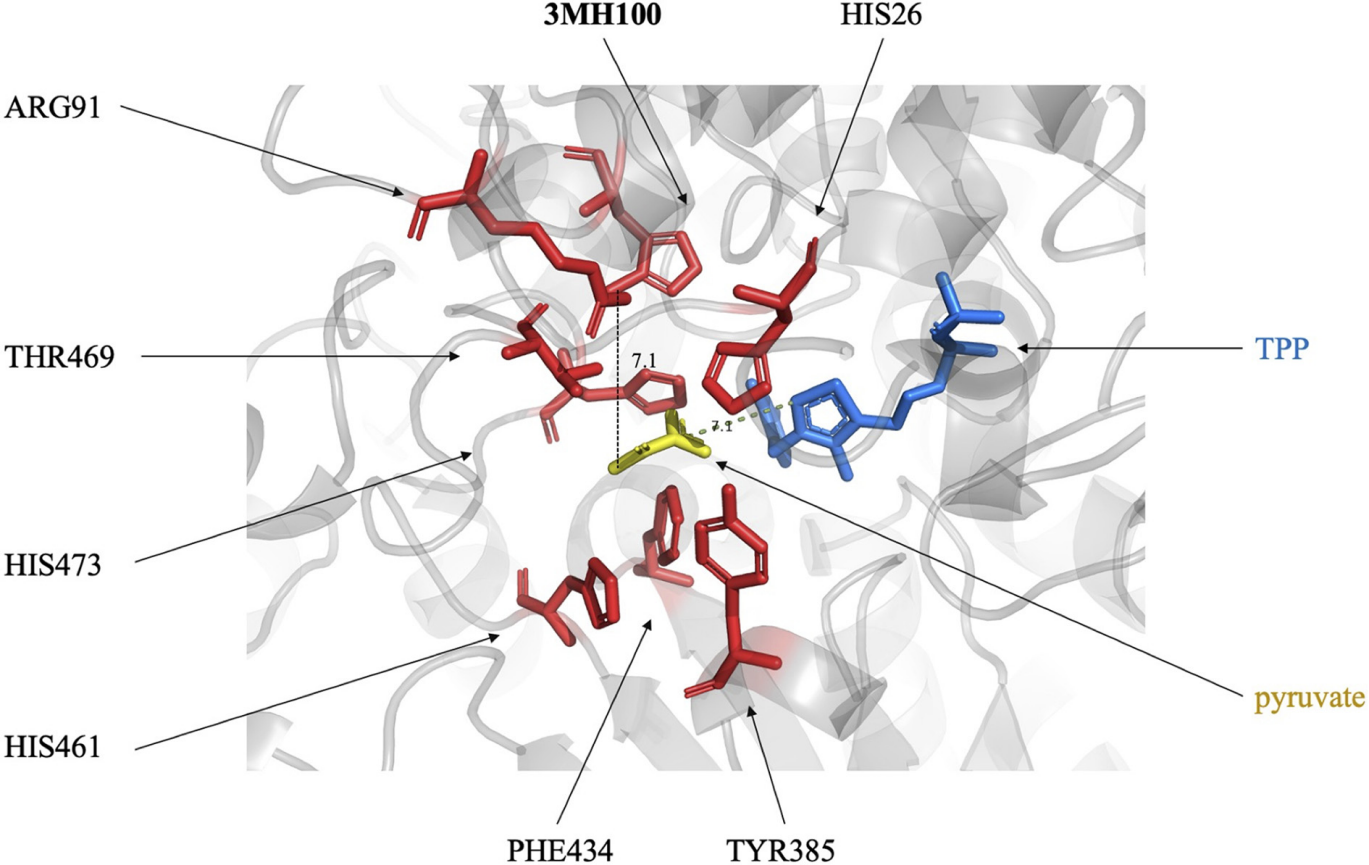
The docking conformation between pyruvate and the H100-3MHI TK variant containing cofactor TPP predicted by Autodock Vina The structure of the H100-3MH TK variant was built on a triple-mutant variant (PDB 5HHT) by Molecular Operating Environment (MOE) [48], energy minimisation was performed by GROMACS in amber99sb-ild forcefield [42]. Pyruvate was drawn in Chem3D-17.0 and energy minimised by MM2 calculations. The active site residues within 4 Å of the substrate are shown as red sticks, donor substrate pyruvate, and cofactor TPP are shown as yellow and blue sticks, respectively. The distance between methyl group on 3MH100 and pyruvate is shown as black-dashed line (7.1 Å), between pyruvate and cofactor TPP is shown as yellow-dashed line (7.1 Å).

Histidine is an essential amino acid, present in the active site of countless enzymes due to the ability of the imidazole side chain to act as a general acid and general base in catalysis, or to chelate metal ions such as in metalloproteins [33]. So far, about 20 analogues of histidine have been reported to be incorporated into proteins [34–37]. A mutant of pyrrolysyl-tRNA synthetase (PylRS – N346A/C348A) can recognise two of the most widely used histidine mimetics [37], 3-methylhistidine (3MH) and thiazole alanine (TA). Replacement of three active-site histidine residues in alanine racemase with 3MH and TA, led to a dramatic change in catalytic efficiency [37]. Similar results were obtained when a histidine in the substrate binding pocket of TK was replaced by 3MH. By analysing the crystal structures of TK complexed with donor substrates, the previous studies showed H100 of *E. coli* and yeast TK forms hydrogen bonds with the C1-hydroxy groups of its donor substrates including D-xylulose-5-phosphate (X5P) and D-fructose-6-phosphate (F6P) [38–40], and so switching the substrate to pyruvate would remove this hydrogen bond due to the lack of the hydroxyl group. Thus, we conjectured that by adding a methyl group to H100, its hydrophobic interaction with pyruvate may be enhanced, thus allowing the substrate to be anchored. Briefly, by reassigning the sense codon at position H100 of *E. coli* TK to an amber codon, the original histidine was successfully replaced by 3MH with the help of an evolved *Mb*PylRS/tRNA^Pyl^ pair (PylHRS; L270I, Y271F, L274G, C313F, and Y349F) [35]. However, the results showed a decrease in activity (unpublished data). The docking conformation of pyruvate with H100-3MH TK containing cofactor thiamine pyrophosphate (TPP) predicted by Autodock Vina [41] indicated that the methyl group on 3MH may have become too distant from pyruvate for hydrophobic interaction to occur. Combining the docking conformation and a molecular dynamics simulation in GROMACS [42], the addition of the methyl group also appeared to induce a conformational change in the surrounding amino acids, which ultimately shifted the pyruvate away from TPP, and so leading to a decrease in activity (Figure 3, unpublished data).

The metal-chelating ligands in metalloenzymes are extremely important and the introduction of nnAAs into key ligand sites can dramatically alter their catalytic properties [43,44]. It was reported that N_δ_-methyl histidine was incorporated into several metalloenzymes to create augmented properties and novel activities [45]. For instance, N_δ_-methyl histidine installed as the proximal heme ligand in the oxygen-binding protein, myoglobin (Mb), can cause a dramatic increase in heme redox potential and promiscuous activity [46]. Similarly, the incorporation of the same nnAA into a modified ascorbate peroxidase resulted in improved robustness towards irreversible deactivation [47].

In addition to the examples above, enzymes engineered with analogues of tyrosine [11,49], proline [50], tryptophan [51], and lysine [52,53] have also been shown to improve catalytic properties. In a functional Mb-based oxidase, replacement of the conserved tyrosine with a series of Tyr analogues with reduced pKa in the phenolic ring demonstrated enhanced activity due to increased protonatability of Tyr [54]. In general, the substitution of amino acids with analogues at the active sites of enzymes has become an ideal method for fine-tuning enzyme activity. It is particularly advantageous that analogues can provide both a higher ‘atom level’ resolution and wider range of modifications to side chain chemistry in terms of sterics, electronics and noncovalent bonding, than the canonical 20 amino acids, as these are more likely to be compatible with the modified site, while also enabling large changes in catalytic properties.

### Improve the stability of enzymes

Stability is a fundamental property of enzymes and has significant impact on their structure, expression, solubility, and function [55,56]. The stability of proteins is due to a combination of factors such as the packing of the hydrophobic core, the level of burial in the hydrophobic surface region, the stability of secondary structure elements, the number of hydrogen bonds, and salt bridges on the protein surface, etc. [57]. The abundant range of side chains of nnAAs certainly provides more options for engineering these factors with the aim of improving the stability of enzymes.

For dimeric enzymes, the use of nnAAs can strengthen the interaction between the monomers and improve the stability of the enzyme. In the research conducted by Schultz group in 2018, the substitution of Phe 21 with *p*-benzoylphenylalanine (pBzF **4**) in *E. coli* homoserine O-succinyltransferase (metA) increased the melting temperature by 21 °C [58]. This may result from the formation of a covalent adduct between the keto groups of Cys 90 and pBzF, which stabilises the dimeric structure of the enzyme. Extensively fluorinated (fluorous) amino acids at dimeric interfaces also seem particularly effective in increasing protein stability [59]. Substitution of a phenylalanine with *p*-fluorophenylalanine (*p*FF **11**) successfully increased the refoldability and thermoactivity of S5 phosphotriesterase (PTE) [60]. Similarly, the *T*_m_ increased by 19 °C in glutathione transferase when all four tryptophan residues in each monomer were replaced with 5-fluorotryptophan [61]. However, of the many members of the fluorinated amino acid family, is it true that the greater the fluorination the more effective it is in enhancing stability? In 2008, three phenylalanine-fluorinated analogues: pentafluoro-L-phenylalanine (F_5_F **22**), 2,3,4,5-tetrafluoro-L-phenylalanine (F_4_F **21**), and 2,3,5,6-tetrafluoro-L-phenylalanine (Zp) were incorporated into the villin headpiece subdomain (HP35) to investigate the relationship between the degree of fluorination and stability. The results showed that Phe10F_4_F had a melting temperature that was 14 °C higher than that of WT, while Phe10F_5_F was only 2 °C higher. This strongly suggested that the **highly but incompletely** fluorinated side chains could provide enhanced hydrophobicity while retaining the ArH…π interactions, thus providing a stable protein fold [62].

**Figure 4:**
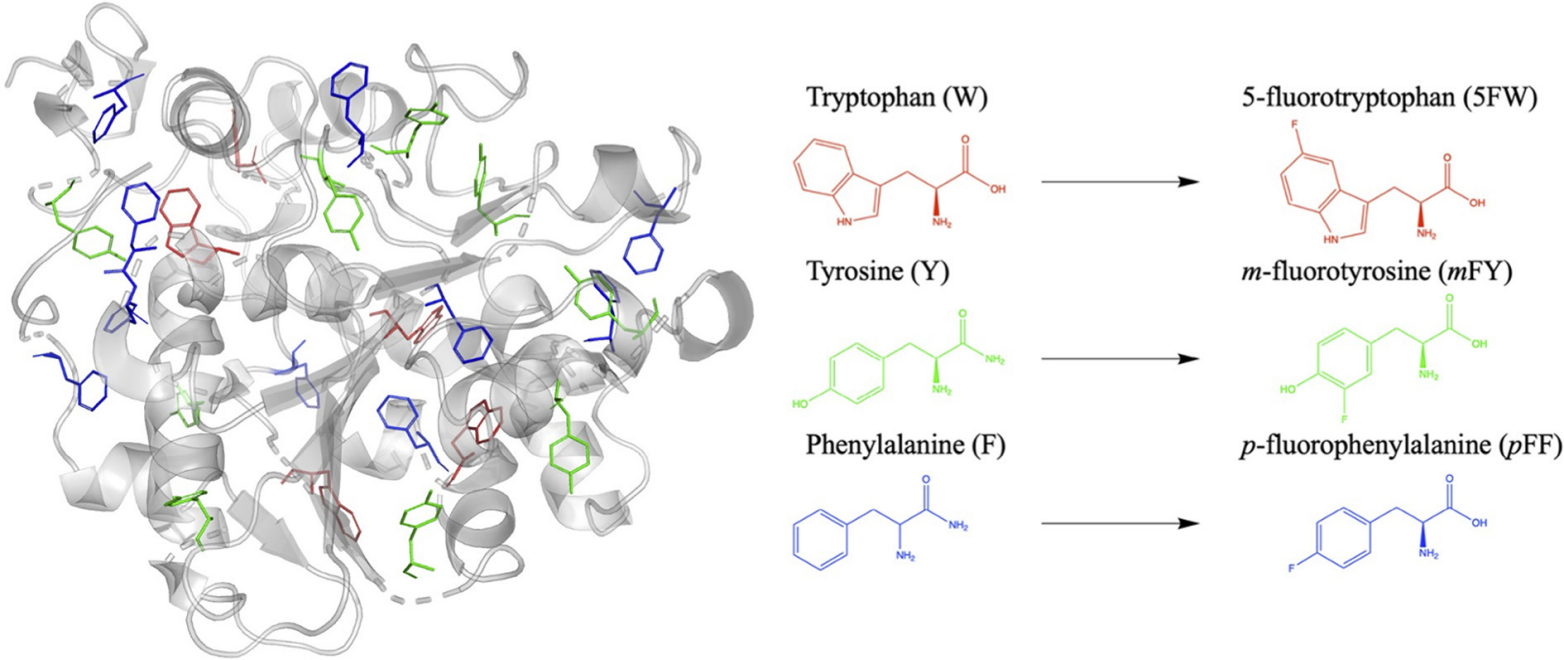
The fluorination of aromatic amino acid residues in *Candida antarctica* lipase B N74D The structures of tryptophan, tyrosine, and phenylalanine in the original PDB structure model (PDB 1TCC) are shown in red, green, and blue sticks, respectively. They were fluorinated experimentally to 5-fluorotryptophan (5FW), *m*-fluorotyrosine (*m*FY), and *p*FF, respectively.

Compared with site-specific substitution, residue-specific incorporation enables global incorporation of nnAAs, which is widely used as a strategy for obtaining mutants with desired properties [63]. In general, residue-specific incorporation of nnAAs can be achieved by multiple biochemical methods in addition to genetic code extension techniques, as the latter is currently inefficient on its own for incorporating nnAAs at multiple sites. A commonly used approach for global fluorination is to supply appropriate nnAAs in auxotrophic strains during expression of desired proteins. By applying this method, all tryptophan, tyrosine, and phenylalanine residues in a glycosylation-deficient mutant of *Candida antarctica* lipase B were substituted by 5-fluoro-L-tryptophan, *o*-fluoro-(DL)-tyrosine, and *p*-fluoro-L-phenylalanine **11** (Figure 4), respectively [64]. This global fluorination prolonged the shelf life of the lipase activity, which is an essential beneficial feature for storage. Following the same strategy, when all prolines were substituted with fluorinated analogues in GFP, the tendency of *cis-trans* isomerisation of the Pro residue changed, and therefore the stability of protein. In comparison, site-specific substitution through genetic encoding has minimal disturbance to the structure. Both methods have their advantages and limitations, thus they should be chosen appropriately according to the purpose.

In addition to activity and stability, the use of nnAAs to improve other enzymatic properties, including stereoselectivity, substrate specificity, reversibility, and immobilisation capacity have also been extensively reported [65,66], and details will not be discussed in the present review. Overall, the emergence of unnatural amino acids has given a new lease of life to enzyme engineering and holds great prospects for the future.

### Optical control of enzymes

The precise spatial and temporal control of enzymes has always been a hot topic. Compared with chemical activation methods, the use of light as an external trigger minimises adverse effects and is therefore of wide interest. The genetic encoding of nnAAs with light-sensitive groups enables the control of a wide range of biological processes *in vivo* and *in vitro* [67]. For instance, by replacing the sulfhydryl and hydroxyl groups of cysteine, serine, and tyrosine with nitrobenzyl groups, photomodulation of proteins can be achieved by the decaging of protecting groups under 365 nm light. To date, the main types of nnAAs that can be inserted into the enzyme to act as protectors are caged lysines [68,69], caged tyrosines [70,71], photoswitchable phenylalanine derivatives [72,73], caged cysteines [74], and selenocysteines [75,76].

**Figure 5:**
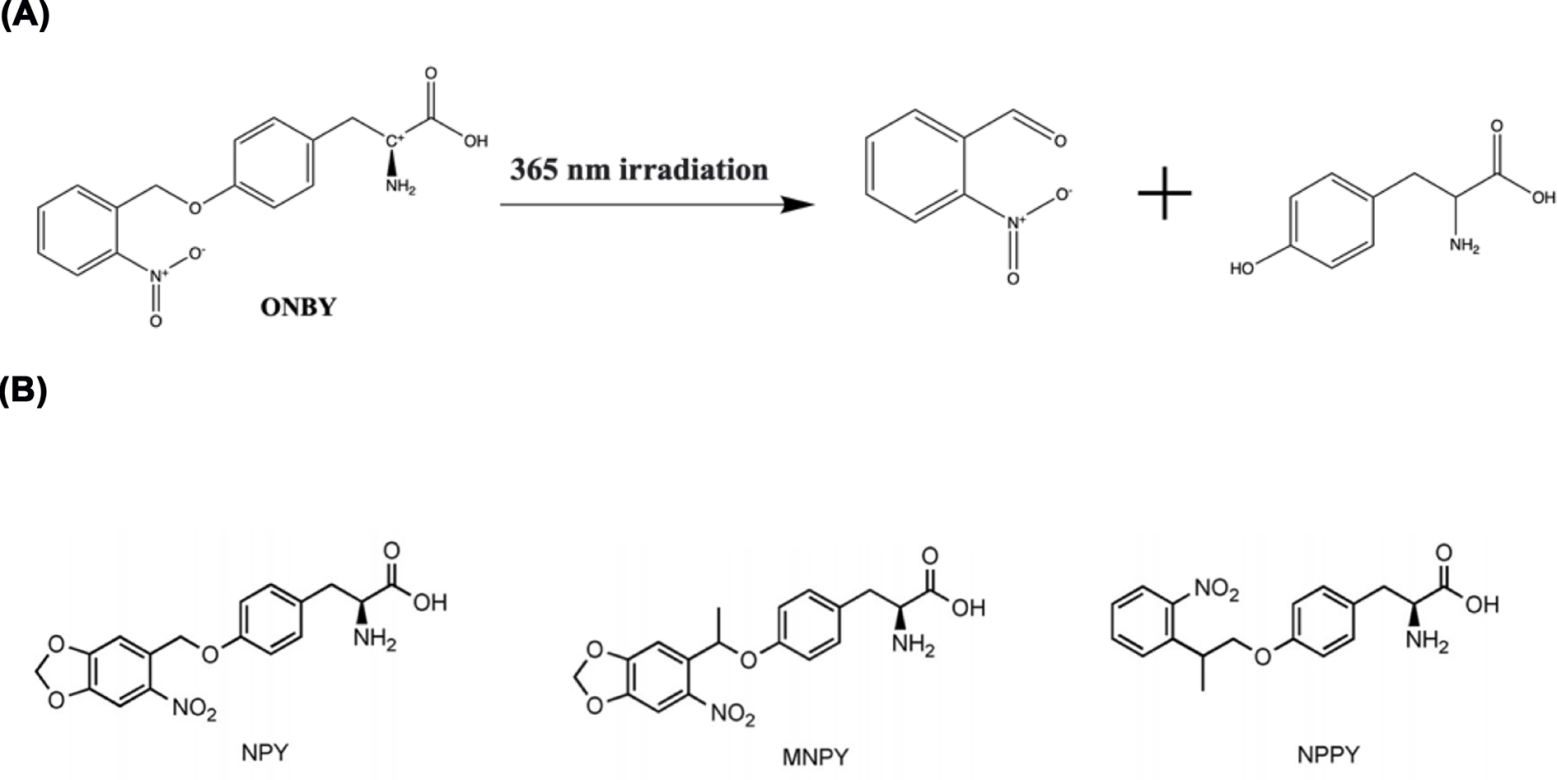
Use of caged-tyrosine analogues (**A**) The deprotected process for OBNY. (**B**) The structure of o-nitropiperonyltyrosine (NPY), MNPY, and NPPY.

Among all photocaging groups, nitrobenzyl groups and their derivatives are the most prestigious, and have been utilised in enzymes through a variety of evolved incorporation systems (Table 1). In the catalogue of tyrosine analogues, the most used is *o*-nitrobenzyltyrosine (ONBY) (Figure 5A). A number of studies have focused on designing enzymes with ONBY to achieve optical control of them [71,77–81]. One example is the creation of light-activated DNA and RNA polymerases. By rational design, ONBY was used to substitute Y61 in the Thermus aquaticus (*Taq*) DNA polymerase active site, where its *o*-nitrophenyl group impedes the extensive mechanical movement of the O-helix and finger-like structural domains, also interferes with the ability to accommodate dNTPs. Normal function of the *Taq* polymerase can be easily restored with the shedding of the protecting group by a 5-min exposure to 365-nm UV light [79]. Following the same strategy, a light-activatable bacteriophage T7 RNA polymerase (T7RNAP) was further generated. The light regulation of enzyme is achieved by removing the *o*-nitrophenyl group to allow the incoming nucleotides to reach the active site of the enzyme [81]. Moreover, the use of ONBY has had an impact in gene-editing. Zinc-finger nucleases (ZFN) have been developed as an important tool for gene editing due to their function in sequence-selective dsDNA cleavage. Photoactivated ZFN would allow conditional generation of gene editing with unprecedented accuracy while tackling toxicity issues [82].

**Table 1:** Summary for caged tyrosines and the responding aaRS

nnAA	aaRS	Protein	Strain	Reference
ONBY	ONBTyrRS	Sperm-whale myoglobin	DH10B *E. coli*	[71]
		β-galactosidase	DH10B *E. coli*	[71]
	BpaRS	*Taq* DNA polymerase	BL21 *E. coli*	[79]
		T7 RNA polymerase	BL21 *E. coli*	[81]
	*Mj*YRSONBY	Zinc-Finger nuclease	BL21 *E. coli*, HEK 293T	[82]
		Imidazole glycerol phosphate synthase	BL21 *E. coli*	[85]
	ONBYRS	Superfold green fluorescent protein	DH10B *E. coli*, HEK 293T	[77]
	ONB-DOPARS-1	3,4-Dihydroxyphenyl-alanine (DOPA)-rich mussel adhesive proteins (MAPs)	B-95.△A *E. coli*	[80]
	ONBYRS-1	Superfold green fluorescent protein	BL21(DE3) *E. coli*	[88]
	NPYRS	Mutated firefly luciferase	HEK 293T	[78]
NPY	NPYRS	Mutated firefly luciferase	HEK 293T	[78]
		TEV protease	HEK 293T	[78]
	*Mj*YRSONBY	Imidazole glycerol phosphate synthase	BL21 *E. coli*	[78]
MNPY	NPYRS	Mutated firefly luciferase	HEK 293T	[78]
		TEV protease	HEK 293T	[78]
NPPY	NPYRS	Mutated firefly luciferase	HEK 293T	[78]
		TEV protease	HEK 293T	[78]

NPY, o-nitropiperonyltyrosine.

Although ONBY has demonstrated its strength in generating photoactivated proteins, it has been noted that *o*-nitrobenzyl has an absorption maximum at 254 nm, resulting in a ‘blue shift’ from the commonly used 365-nm light source. In contrast, the three other caged tyrosine analogues, namely methyl *o*-nitropiperonyltyrosine (MNPY), *o*-nitropiperonyltyrosine (NPY) and *o*-nitrophenylpropyltyrosine (NPPY) (Figure 5B) showed better photochemical properties. Their incorporation into a protease demonstrated the ability of off-to-on switching [78]. However, due to the complex synthetic routes, low incorporation efficiency and fidelity, these derivatives are far less applicable than ONBY.

Carrying the same nitrobenzyl-derivative groups, caged lysines have also been used to replace lysine residues in numerous enzymes including kinases [83], Cre recombinase [70,84], Cas9 nuclease [84], isocitrate dehydrogenase [85], imidazole glycerol phosphate synthase [86], and so forth to achieve optical control. Intriguingly, spontaneous decaging of nitrobenzene groups and their derivatives in *E. coli* in the absence of UV irradiation has also been reported (with the decaging ratio of 24–85%), possibly due to bacterial NTR-mediated elimination [87]. Furthermore, bulky side chains of caged nnAAs make the selection of insertion sites challenging. Thus, although nitrobenzyl groups have demonstrated their ability to optically control proteins *in vivo* and *in vitro*, its effectiveness in decaging varies widely in reports and remains as a challenge to be addressed.

#### nnAAs in enzymes as probes

In addition to the applications mentioned above, enzymatic engineering with the participation of nnAAs also demonstrates its benefits in an ever-increasing range of aspects as the variety of available sidechains increases. Some nnAAs with functional groups can be incorporated into enzymes as probes, which play important roles in the characterisation of protein structures, protein interactions, and the detection of protein dynamics. In general, nnAAs probes can be classified into the following categories: (1) infrared (IR) and NMR probes, (2) photocross-linkers, (3) probes with heavy atoms, (4) fluorescent probes, and (5) spin-label probes [89].

Fluorescence approaches have been widely used to elucidate the dynamics of proteins. However, global, nonspecific fluorescence often alters the natural structure of proteins. The site-specific insertion of nnAAs-carrying fluorescent moieties is a promising way to overcome this challenge. For instance, since the incorporation system of L-3-(6-acetylnaphthalen-2-ylamino)-2-amninopropanoic acid (ANAP) was first evolved in 2003, it has been widely used as a fluorescent probe in eukaryotic and prokaryotic systems, benefiting from its small size and high sensitivity [90]. The dynamics of numerous proteins, including luciferase [91], GFP, and voltage-sensing phosphatases [92], have been revealed by the nature of ANAP’s spectral redshift in response to environmental polarisation. It has also shown great ability in depicting the structure of multiple channels and gates, which includes but is not limited to KCNH channels [93], CNGA1 channels [94], ASIC channels [95], TRPV1 channels [96], and Nav1.5 inactivation gates [92].

The click reaction, copper(I)-catalysed alkyl-azide cyclisation (CuAAC, click reaction) is another classical method for labelling [97]. The introduction of nnAAs containing azide or alkynes allows labelling of an increasing number of proteins, including epidermal growth factor receptor (EGFR) [98], *E. coli* outer membrane protein C (Omp C) [99], mannitol dehydrogenase (MDH), and formate dehydrogenase (FDH) [100]. However, the cytotoxicity caused by the Cu(l) metal catalyst limited its application in living cells. To enable a broader application in bioorthogonal labelling, numerous strategies including strain-promoted Cu(I)-free azide-alkyne cycloaddition (SPAAC), chelation-assisted CuAAC, and ligand-assisted CuAAC have been developed [101].

**Figure 6:**
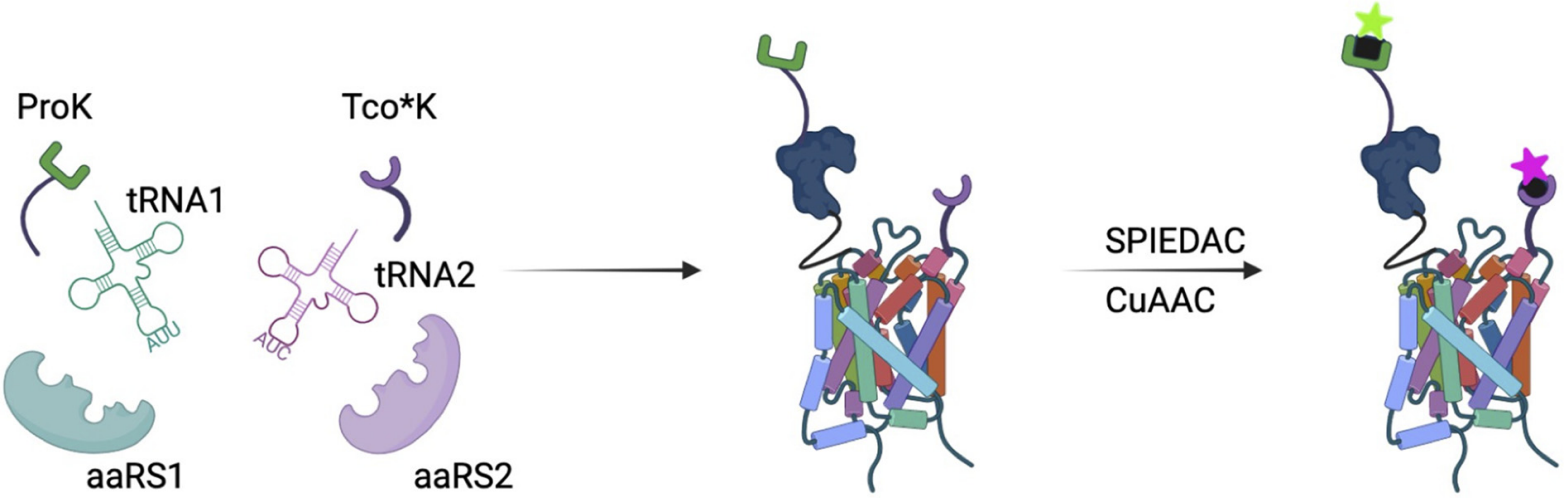
Scheme illustrating dual incorporation of Tco*K and ProK into a receptor and the subsequent dual bio-orthogonal labelling

Furthermore, dual labelling has become feasible due to the emergence of multiple nnAA incorporation systems, which is especially useful in Förster resonance energy transfer (FRET) experiments. The reported coupled nnAAs include *p*-azidophenylalanine (pAzF)/N6-((2-propynyloxy) carbonyl)-L-lysine [102], *p*-acetylphenylalanine/alkynyllysine [103], *p*AzF/Tet3.0 [104], 4-biphenyl-L-phenylalanine/L-(7-hydroxycoumarin-4-yl)-ethylglycine [105], and have been successfully employed to illustrate the dynamic changes in a wide range of proteins. An elegant example of this is the dual site-specific fluorescent labelling toolbox developed by Meineke *et al.* By combining *trans*-cyclooct-2-ene-L-lysine (TCO*K) and *N*-Propargyl-L-lysine (ProK) incorporation with CuAAC/SPIEDAC, the labelling of Notch receptors and a G protein-coupled receptor (GPCR) has been achieved for the first time in living cells (Figure 6). These two bio-orthogonal reactions allow precise control and direction for probing and manipulation of proteins in space and time, and therefore hold promise for a wide range of applications [106].

## Designing enzymes with nnAAs *in silico*

Advances in computational technology have greatly facilitated the identification of hotspots for enzyme modification, including active-site residues, access tunnel sites, flexible sites, interface sites, etc. [28]. In particular, with the help of molecular modelling software such as GROMACS [42], CHARMM [107], AMBER [108], and FoldX [109], numerous studies have been carried out on enzymes to investigate their biochemical reactivity, thermodynamics, and interactions with substrates and solvents. However, most of them cannot be applied to simulations of proteins containing nnAAs as few contain parameterisations for unnatural side chains. To achieve simulations of proteins containing a wider variety of nnAAs, manually adding parameters for nonstandard residues obtained from multiple tools is an option, but this is a tedious and time-consuming process. More importantly, tools for autogenerated parameters are not compatible with all force fields and often lack good validation. Therefore, easier, faster, and experimentally validated methods for the *in-silico* introduction of nnAAs have become an urgent need. The good news is that extensive studies have recently provided parameters for many commonly used nnAAs in different force fields (Table 2). The Zagrovic group has developed over 250 post-translational modification parameters on the new GROMOS 54a7 force field, which has been verified to exhibit near-chemical accuracy in measuring hydration-free energies. This high-resolution computational tool greatly extends the range of artificial side chains that can be modelled [110]. Moreover, the Flodas lab released an online tool called Forcefield_NCAA that provides charge parameters for a large library of 147 nnAAs compatible with AMBERff03. Similarly, developed parameters of 18 derivatives of phenylalanine and tyrosine were validated in Amber ff14SB [111]. Recently, more and more nnAAs with specific sidechains such as halogenated groups, azido and alkynyl groups [112] are being parametrised.

**Table 2:** Summary for forcefields modified with nnAAs parameters

Ref. of modified forcefields	Compatible forcefield	Applicable nnAAs
[120]	CHARMM36	406 nnAAs
[121]	CHARMM19	Methylated lysines and arginines, acetylated lysines
[121]	CHARMM127	Methylated lysines and arginines, acetylated lysines
[111]	Amber ff14SB	18 phenylalanine and tyrosine derivatives
[122]	AMBERff03	147 nnAAs
[123]	AMBER ff15ipq	Fluorinated nnAAs
[124]	CHARMM22	13 nnAAs
[125]	CHARMM general force field (CGenFF)	nnAAs with Azido and Alkynyl R‐Groups
[112]	COMPASS force field	nnAAs with Aliphatic Azide Chains
[110]	GROMOS 45a3 and 54a7	250 different types of Post-Translational Modification

Rosetta, a powerful tool for the *ab initio* engineering of enzymes has been applied to design variants with diverse goals [113]. A number of enzymes involved in the Dies-Alder [114], Retro-aldol [115], and Kemp elimination [116] reactions have been designed using this approach and validated experimentally. However, while tools have been designed in Rosetta to handle artificial groups [117–119], the use of Rosetta to design nnAAs-containing enzymes has hardly been applied in practise.

## Challenges and prospects

The examples presented show that genetically encoded nnAAs have certainly become a hot spot in the field of biocatalyst engineering. However, it is of concern that its development is still limited by multiple factors. Firstly, a large number of nnAAs have very complex synthetic and downstream purification routes, which limit their mass production and lead to high costs [126]. This also directly contributes to the difficulty of producing protein products containing nnAAs on a large scale in industry. To overcome this challenge, it is of great importance to develop more cost-effective methods for synthesising nnAAs. Two general routes are chemical synthetic routes and biocatalytic routes. The former often suffers from a lack of enantioselectivity and stereoselectivity [127]. In contrast, biosynthetic routes are highly attractive due to their simple reaction conditions, low energy consumption, and high enantiomeric excess (*e.e.*). For instance, L-tert-leucine is an important precursor for the manufacture of protease inhibitors, which are of great value in clinical applications. The chemical synthesis of L-tert-leucine is limited by low stereoselectivity, low yields, and high pollution. In the development of its biosynthetic method, an evolved leucine dehydrogenase was screened out using a high-throughput spectrophotometric screening assay, resulting in a high yield of 1170 g/day [128].

Another issue of great importance is the incorporation efficiency/fidelity of nnAAs. To date, hundreds of nnAAs have been reported to be genetically encoded, but it is doubtful how many of these can be introduced by orthogonal translation systems at high efficiency and with high fidelity. In the research of our group, a methodology to quantify incorporation fidelity from deconvoluted mass spectra was introduced, and it was used to evaluate the incorporation performance of some of the most commonly used phenylalanine analogues in a specific site of TK [30]. Of these, *p*CNF had a misincorporation rate of around 22%, while it was as high as about 55% for *p*AMF. Although this depends on a range of factors, including protein structure, the specific site of insertion and other elements on the plasmid, it should still place a high priority on the most vital factor: the effectiveness of the orthologous tRNA/synthase pairs. As mentioned above, more than two-thirds of nnAA expression was achieved by adopting TyrRS-tRNA^Tyr^ or PylRS-tRNA^Pyl^ systems, which leads to the result that only analogues of tyrosine and pyrrolysine can be introduced in a relatively efficient manner [6]. To enable the use of a high diversity of artificial residues, more orthogonal translation systems need to be evolved.

In summary, nnAAs have already been extensively used in enzyme engineering. Their powerful functional side chains have conferred new features or improved the properties of numerous enzymes, and there is undoubtedly considerable potential to expand their applications. However, there are still many obstacles to overcome to make this more wide-ranging, efficient, and economical, though we are already on the way.

## Open Access

Open access for the present article was enabled by the participation of University College London in an all-inclusive *Read & Publish* agreement with Portland Press and the Biochemical Society under a transformative agreement with JISC.

## Abbreviations

3-HBA: 3-hydroxybenzaldehyde
3MH: 3-methyl-histidine
aaRS: aminoacyl-tRNA synthetase
ANAP: L-3-(6-acetylnaphthalen-2-ylamino)-2-amninopropanoic acid
CAGR: Compound Annual Growth Rate
CuAAC: copper(I)-catalysed alkyl-azide cyclisation
DOPA: 3,4-dihydroxyphenyl-alanine (DOPA
F4F: 2,3,4,5-tetrafluoro-L-phenylalanine
F5F: pentafluoro-L-phenylalanine
F6P: D-fructose-6-phosphate
FRET: Förster resonance energy transfer
GCE: genetic code expansion
GFP: green fluorescent protein
IR: infrared
Mb: myoglobin
Mj: Methanocaldococcus jannaschii
MNPY: methyl o-nitropiperonyltyrosine
MOE: molecular operating environment
ncAA: noncanonical amino acid
nnAA: non-natural amino acid
NMR: nuclear magnetic resonance
NPPY: o-nitrophenylpropyltyrosine
NPY: o-nitropiperonyltyrosine
NTR: nitroreductase
ONBY: o-nitrobenzyltyrosine
pAzF: p-azidophenylalanine
pBzF: p-benzoylphenylalanine
pCNF: p-cyanophenylalanine
PDB: protein databank
pFF: p-fluorophenylalanine
ProK: N-propargyl-L-lysine
PTE: phosphotriesterase
PylRS: pyrrolysyl-tRNA synthetase
TA: thiazole alanine
TAm: transaminase
Taq: thermus aquaticus
TCO*K: trans-cyclooct-2-ene-L-lysine
TK: transketolase
TPP: thiamine pyrophosphate
UV: ultraviolet
X5P: D-xylulose-5-phosphate
ZFN: zinc-finger nuclease
